# Association between Dietary Phenolic Acids and Hypertension in a Mediterranean Cohort

**DOI:** 10.3390/nu9101069

**Published:** 2017-09-27

**Authors:** Justyna Godos, Dario Sinatra, Isabella Blanco, Serena Mulè, Melania La Verde, Marina Marranzano

**Affiliations:** Department of Medical and Surgical Sciences and Advanced Technologies “G.F. Ingrassia”, University of Catania, 95123 Catania, Italy; sinatradario@gmail.com (D.S.); dott.ssa.blanco.isabella@gmail.com (I.B.); serenamule86@gmail.com (S.M.); melanialaverde@yahoo.it (M.L.V.); marranz@unict.it (M.M.)

**Keywords:** phenolic acids, polyphenols, fruit, vegetable, nuts, hypertension, cohort study, Mediterranean area

## Abstract

Background: Certain foods rich in phenolic acids have been shown to reduce the risk of hypertension, but evidence from epidemiological studies focused on dietary phenolic acid intake is scarce. The aim of this study was to determine the association between dietary phenolic acid intake, as well as their major food sources, and hypertension in a Mediterranean cohort. Methods: Demographic and dietary data of 2044 adults living in Southern Italy were collected. Food frequency questionnaires and Phenol-Explorer were used to calculate dietary intake of polyphenols. Multivariate logistic regression analyses were used to test associations. Results: The mean intake of total phenolic acids in the cohort was 362.6 mg/day. Individuals in the highest quartile of phenolic acid intake (median intake = 522.2 mg/day) were less likely to have hypertension (OR (odds ratio) = 0.68, 95% CI (confidence interval): 0.46, 1.00). When taking into account individual subclasses of phenolic acids, only hydroxyphenylacetic acid was inversely associated with hypertension (highest vs. lowest quartile, OR = 0.63, 95% CI: 0.40, 0.96). Among dietary sources of phenolic acids considered in the analysis, only beer was significantly inversely associated with hypertension (highest vs. lowest quartile, OR = 0.32, 95% CI: 0.15, 0.68). Conclusions: The findings of this study suggest that dietary phenolic acids may be inversely associated with hypertension, irrespectively of their dietary source.

## 1. Introduction

Plant-based dietary patterns have been associated with better metabolic profile and lower risk of non-communicable diseases [1,2,3]. Key components, such as fruits and vegetables, but also tea, have been associated with beneficial effects on health [4]. The potential effects of other foods, such as beer, wine, nuts and coffee, have been historically controversial due to their content of compounds (such as ethanol, fats, and caffeine, respectively) that are known to exert detrimental effects on health. However, their impact on health has been recently reconsidered [5,6,7]: indeed, summary results of epidemiological studies show an overall decreased mortality risk associated with moderate consumption of such foods and beverages [8,9,10]. The biological rationale of this potential benefit may rely on a better knowledge of the role of antioxidants in the diet and potential risk modification depending on the quantity and quality of compounds representative of the aforementioned foods and beverages [11,12].

Dietary polyphenols have been recently studied as potential mediators of the beneficial effects of antioxidant-rich foods on certain health outcomes. With regard to anti-hypertensive effects, dietary polyphenols have demonstrated antioxidant, anti-inflammatory, and antithrombotic properties, as well as their capacity to affect NO (nitric oxide) bioavailability [13]. Among common classes of polyphenols in tea, nuts, coffee, olive oil, and alcoholic beverages, phenolic acids are compounds containing a phenolic ring and an organic carboxylic acid function (C6-C1 skeleton) [14]. Observational studies on the association between phenolic acids and cardio-metabolic risk factors are scarce. Results from cross-sectional studies are not univocal, as some studies showed no significant findings [15] while others showed a relation between phenolic acid intake and blood pressure as component of metabolic syndrome [16,17]. Only one prospective study including phenolic acid exposure, and specifically assessing hypertension as an outcome, has been published to date, showing a significant lower risk of disease [18]. Overall, evidence on such matters is still limited and more investigations are needed to confirm the consistency of results and better identify potential dietary sources of phenolic acids that may play a role in decreasing the risk of hypertension. Therefore, the aim of this study was to assess the association between dietary phenolic acid consumption, as well as their major food sources, and hypertension in a cohort of adults living in the Mediterranean area.

## 2. Materials and Methods 

### 2.1. Study Design and Population

The MEAL (Mediterranean healthy Eating, Ageing, and Lifestyle) study is an observational study primarily designed to investigate the relationship between nutritional habits characterizing the classical Mediterranean lifestyle and non-communicable diseases. The baseline data included a random sample of the general population including 2044 men and women aged 18 years or older, stratified by gender and 10-year age groups, randomly selected in the main districts of the city of Catania, Southern Italy. The enrollment and data collection was performed between 2014 and 2015 through the selection among the lists of registered patients of a pool of general practitioners. Full details of the study protocol are published elsewhere [19]. All participants were informed about the aims of the study and provided written informed consent. All of the study procedures were carried out in accordance with the Declaration of Helsinki (1989) of the World Medical Association. The study protocol has been approved by the concerning ethical committee.

### 2.2. Data Collection

An electronic data collection was performed by a face-to-face computer-assisted personal interview using tablet computers. In order to visualize the response options, participants were provided with a paper copy of the questionnaire. However, final answers were registered by the interviewer directly on the digital device (tablet computer). The demographic data comprised gender, age at recruitment, latest educational degree achieved, occupation (specifies the character of the most important employment during the year before the investigation) or last occupation before retirement, and marital status; educational status was categorized as (i) low (primary/secondary); (ii) medium (high school); and (iii) high (university). Occupational status was categorized as (i) unemployed; (ii) low (unskilled workers); (iii) medium (partially-skilled workers); and (iv) high (skilled workers) [20]. Physical activity status was evaluated through the International Physical Activity Questionnaires (IPAQ) [21], which included a set of questionnaires (5 domains) investigating the time spent being physically active in the last seven days: based on the IPAQ guidelines, final scores allow the physical activity level to be categorized as (i) low; (ii) moderate; and (iii) high. Smoking status was categorized as (i) non-smoker; (ii) ex-smoker; and (iii) current smoker. Alcohol consumption was categorized as (i) none; (ii) moderate drinker (0.1–12 g/day); and (iii) regular drinker (>12 g/day).

### 2.3. Dietary Assessment

The dietary assessment was performed by the administration of two food frequency questionnaires (a long and a short version) that were previously tested for validity and reliability for the Sicilian population [22,23]. Subsequently, the identification of the food intake, the energy content, as well as the macro- and micro-nutrient intake were obtained through comparison with food composition tables of the Research Center for Foods and Nutrition [24]. Intake of seasonal foods referred to consumption during the period in which the food was available and then adjusted by its proportional intake in one year. FFQs (Food Frequency Questionnaires) with unreliable intakes (<1000 or >6000 kcal/day) were excluded from the analyses (*n* = 107) leaving a total of 1937 individuals included in the analysis. 

### 2.4. Estimation of Polyphenol Intake

The process of the estimation of polyphenol intake was described in detail elsewhere [25]. Briefly, data on the polyphenol content in foods were obtained from the Phenol-Explorer database (www.phenol-explorer.eu) [26]. A new version of the Phenol-Explorer database containing data on the effects of cooking and food processing on polyphenol contents was used whenever possible in order to apply polyphenol-specific retention factors [27]. Foods that contained no polyphenols were excluded from the calculation, leaving a total of 75 items included in the analyses. Weight loss or gain during cooking was corrected using yield factors [28]. The average food consumption was calculated (in g or mL) by following the standard portion sizes used in the study and then converted in 24-h intake. Finally, a search was carried out in the Phenol-Explorer database to retrieve mean content values for all polyphenols contained in the selected foods. Next, polyphenol intake from each food was calculated by multiplying the content of each polyphenol by the daily consumption of each food. The main classes of polyphenols (flavonoids, phenolic acids, lignans, stilbenes, others) and the total polyphenol intake was estimated by the sum of the previous; additional subclass and selected individual polyphenols were also estimated. Finally, total and individual classes of polyphenol intake were adjusted for total energy intake (kcal/day) using the residual method [29]. 

### 2.5. Anthropometric Measurements and Outcome Ascertainment

Height was measured to the nearest 0.5 cm without shoes, with the back square against the wall tape, eyes looking straight ahead, with a right-angle triangle resting on the scalp and against the wall. Waist circumference (centimeters) was measured midway between the 12th rib and the iliac crest. Body mass index (BMI) was calculated, and patients were categorized as under/normal weight (BMI < 25 kg/m^2^), excess body weight (BMI ≥ 25 kg/m^2^), and obese (BMI > 30 kg/m^2^).

Arterial blood pressure was measured in sitting position and at least 5 min at rest at the end of the physical examination. Because of the possibility of differences in blood pressure measurement, the measurements were taken three times at the right arm relaxed and well supported by a table, with an angle of 45° from the trunk. A mean of the last two measurements was calculated and considered for inclusion in the database. Information from measurements was integrated with general practitioners’ computerized records, as patients are diagnosed with disease by a specialist in order to obtain drug reimbursement. Patients were considered hypertensive when average systolic/diastolic blood pressure levels were higher or equal to 140/90 mm Hg, taking anti-hypertensive medications, or being previously diagnosed with hypertension.

### 2.6. Statistical Analysis

Frequencies are presented as absolute numbers and percentages; continuous variables are presented as means and standard deviations. Individuals were divided into quartiles of phenolic acid intake and the distribution of background characteristics were compared between groups. Differences were tested with the Chi-square test for categorical variables, ANOVA for continuous variables distributed normally, and the Kruskall-Wallis test for variables not distributed normally. Age- and energy-adjusted multivariate logistic regression models were used to test the association between variables of exposure (including total phenolic acids and individual subclasses and specific compound intake) and having hypertension; additional multivariate models adjusted for all other background characteristics (age, sex, BMI, educational and occupational status, smoking and alcohol drinking habits, physical activity level, and dietary sodium, potassium, calcium, and magnesium intake) were also performed to test whether the association retrieved was independent from the aforementioned potential confounding factors. Finally, an additional analysis on the association between major food sources of phenolic acids and hypertension was finally performed based on the results of a previous study in which they were identified as coffee, nuts, tea, olive oil, red wine, white wine, and beer [25]. All reported *p*-values were based on two-sided tests and compared to a significance level of 5%. SPSS 17 (SPSS Inc., Chicago, IL, USA) software was used for all the statistical calculations.

## 3. Results

Baseline characteristics of 1936 participants included in the analysis according to quartiles of phenolic acid intake are shown in Table 1. Among individuals in the highest category of phenolic acid intake there was a higher percentage of regular and moderate drinkers, current and ex-smokers, and individuals with lower educational and occupational level (higher percentage of unemployed) (Table 1). No further significant differences were found regarding sex, age, and physical activity (Table 1). With increasing intake of phenolic acids, a higher intake of micronutrients that might affect blood pressure (including sodium, potassium, calcium, and magnesium) was observed; in contrast, adherence to the Mediterranean diet did not follow a linear association with intake of phenolic acids, as a higher percentage of individuals’ high adherence to this dietary pattern had intermediary consumption of phenolic acids (Table 1). All other major macro- and micronutrients were associated in a direct manner with intake of phenolic acids, suggesting a wide distribution in foods commonly consumed by participants not related to diet quality features (as identified with the Mediterranean diet) (Supplementary Table S1). 

The mean intake of phenolic acids in the cohort was 362.6 mg/day; when considering the most adjusted model (model 3), individuals in the highest quartile of total phenolic acid intake (median intake = 522.2 mg/day) were less likely to have hypertension (OR (odds ratio) = 0.65, 95% CI (confidence interval): 0.43, 0.98) despite there was no clear trend across quartiles of exposure (Table 2). The analysis of individual subclasses of phenolic acids and specific compounds revealed a significant association with hypertension only of hydroxycinnamic acid (highest vs. lowest category, OR = 0.52, 95% CI: 0.32, 0.95) and hydroxyphenylacetic acids (highest vs. lowest category, OR = 0.61, 95% CI: 0.39, 0.97). When analyses were stratified by sex, a significant inverse association between hydroxycinnamic and hydroxyphenylacetic acids and hypertension was found in women, but not in men, while an inverse association with caffeic acid was found in men (Supplementary Table S2).

Among major dietary sources of phenolic acids considered in this study, only beer was significantly inversely associated with hypertension (highest vs. lowest category, OR = 0.32, 95% CI: 0.15, 0.68; Table 3).

## 4. Discussion

In this study, the relation between phenolic acid intake and hypertension was assessed in a cohort of adults living on a Mediterranean island. Dietary intake of phenolic acids in the highest quartile (roughly >400 mg/day) was inversely associated with hypertension; among the main classes and individual compounds investigated, hydroxybenzoic, and hydroxyphenylacetic acids showed a linear inverse association with having hypertension. When considering major dietary sources of phenolic acids, no specific food showed significant association with hypertension besides beer intake.

Observational studies exploring the association between phenolic acids and hypertension are scarce. In a cross-sectional study conducted in 550 adults living in Brazil the highest intake of phenolic acids was not associated with having hypertension, but the middle category of exposure (about 300 mg/day) showed an inverse association [16]. Another cross-sectional study conducted on 2618 Iranian adults showed no association between phenolic acid intake and hypertension as a component of metabolic syndrome; however, intake reported in this study was particularly low compared to the others (median intake of the highest category of exposure was 70.7 mg/day), suggesting that a lower number of food sources might have been used and, thus, data might not be reliable [15]. In contrast, a cross-sectional study conducted on 8821 men and women living in Poland showed that individuals with higher intake of phenolic acids were less likely to have impaired blood pressure (as component of the metabolic syndrome) compared to those with lower intake [17]; interestingly, a prospective study conducted on a subgroup of individuals belonging to the same cohort (those without hypertension on baseline), confirmed the previous findings, despite the results being significant only among women [18].

Among others, the compounds proposed to be responsible for beneficial effects on endothelium are phenolic acids [30,31]. Phenolic acids have been reported to improve endothelial health, and protect against atherothrombosis or atherosclerotic lesion development through attenuation of oxidative stress [32]. The hypotensive properties of phenolic acids have been hypothesized to depend on several mechanisms, such as a NO-mediated vasodilatatory effect, the attenuation of oxidative stress (reactive oxygen species) by reducing NAD(P)H-dependent (nicotinamide adenine dinucleotide phosphate) super-oxide production, and the interaction with the renin–angiotensin aldosterone system by inhibiting angiotensin-converting enzyme activity [33,34]. These compounds may also exert antioxidant effects by scavenging or neutralizing reactive oxidant species and building endogenous antioxidant defenses by down-regulating inflammatory genes in endothelial cells and macrophages [35].

In the present study, only hydroxycinnamic and hydroxyphenenlacetic acids were associated with hypertension. It may be hypothesized that the retrieved associations between consumption of certain foods and blood pressure may be mediated, at least in part, by their content in phenolic acids. However, among major sources of hydroxycinnamic acids, coffee did not show an independent association with blood pressure levels, despite recent evidence showed a decreased risk of hypertension in cohort studies [36,37]. In contrast, a significant inverse association between beer consumption (which was the major source of hydroxyphenenlacetic acids) and hypertension was found; despite heavy alcohol intake being associated with a higher risk of hypertension [38], a large amount of scientific literature agrees that moderate consumption of alcoholic beverages may exert beneficial effects on human health [39,40]. Meta-analyses of prospective cohort studies showed decreased risk of mortality of cardiovascular causes for moderate consumption of alcohol [41]. The significant reduction in vascular risk with a J-shaped relation associated with wine and beer consumption indicated a comparable protecting effect of either beverage potentially due to their common components, such as polyphenols. Around 30% of polyphenols from beer are derived from hops and 70–80% originates from malt [42]; the structural classes of polyphenols in beer include phenols, and benzoic and cinnamic acid derivatives, including hydroxyphenylacetic acid, which was inversely associated with hypertension in the present study.

Some of the food groups investigated in this study are characteristic of healthy dietary patterns, such as the Mediterranean diet. The effect of nut and olive oil consumption on blood pressure has been investigated within the context of healthy diets showing significant results [43,44,45,46,47,48]. Clinical studies showed that moderate consumption of wine and beer as part of the Mediterranean diet had beneficial effects toward biomarkers of hypertension [49,50], especially among fast ethanol metabolizers [51]. Recent evidence suggests that the beneficial effects of the Mediterranean diet may depend on their content in polyphenols [52,53,54,55]. The Mediterranean dietary pattern has been associated with lower risk of cardio-metabolic disorders [56,57,58,59,60,61]. Previous results from this cohort showed a significant inverse association between higher adherence to this dietary pattern and hypertension [62]; however, another study showed that phenolic acid intake was associated in a non-linear manner to high adherence to the Mediterranean diet, suggesting that certain food sources of such polyphenol group not included in the classical paradigm of key features of the Mediterranean diet may provide beneficial action toward cardiovascular health [63]. Additionally, other studies conducted on a similar population (a sample recruited in the same southern Italian area) reported that food groups not included in the Mediterranean diet (i.e., coffee and beer) were associated with better metabolic health [64,65,66]. Overall, adherence to the Mediterranean dietary pattern might be an optimal choice for preventing non-communicable chronic diseases; moreover, the inclusion of certain foods generally not contemplated in this dietary pattern (such as coffee or beer) may improve the aforementioned potential benefits [67].

The findings of the present study should be considered in light of some limitations. The study design was cross-sectional, thus, we estimated the strength of association between the variables of interest and hypertension, but we are unable to define causal relation. Such a study design may be associated with reverse causation, with individuals affected by disease being less likely to have a higher intake of certain foods (i.e., coffee). However, the study was rather focused on phenolic acids, which may have several dietary sources and showed more consistent results than individual foods. Second, questions of FFQ on nut consumption did not refer to salt content (i.e., salted peanuts), thus, we are unaware whether nuts consumed were actually salted. However, in our analyses we adjusted for micronutrients related to salt intake in order to limit the potential confounding effect; moreover, whether confounding exists, it would weaken, rather than strengthen, the association between nut consumption and hypertension.

## 5. Conclusions

In conclusion, the findings of this study suggest that dietary phenolic acids may be inversely associated with hypertension, irrespective of their dietary source. Thus, phenolic acid content of the diet may be responsible for the observed beneficial effects on blood pressure of certain foods. Dietary intervention programs promoting increased intake of phenolic acids through higher consumption of functional foods are needed to confirm the promising findings obtained from this study. More in-depth laboratory studies are also needed to corroborate the exact mechanisms of action of such foods in order to better understand which compounds improve endothelial health and may affect the risk of hypertension.

## Figures and Tables

**Table 1:** Background characteristics by quartiles of dietary phenolic acids in the MEAL study sample (*n* = 1936); Q = quartile.

	Phenolic Acid Intake				*p*
	Q1 (Median = 120.36)	Q2 (Median = 205.39)	Q3 (Median = 307.70)	Q4 (Median = 522.26)	
Sex, *n* (%)					0.919
Male	186 (41.1)	197 (40.5)	215 (42.3)	206 (42.2)	
Female	267 (58.9)	290 (59.5)	293 (57.7)	282 (57.8)	
Educational level, *n* (%)					<0.001
Low	150 (33.1)	161 (33.1)	198 (39.0)	188 (38.5)	
Medium	141 (31.1)	218 (44.8)	177 (34.8)	184 (37.7)	
High	162 (35.8)	108 (22.2)	133 (26.2)	116 (23.8)	
Occupational level, *n* (%)					0.046
Unemployed	94 (22.8)	110 (27.0)	134 (29.7)	123 (31.9)	
Low	69 (16.7)	75 (18.4)	67 (14.9)	55 (14.2)	
Medium	111 (26.9)	95 (23.3)	131 (29.0)	103 (26.7)	
High	139 (33.7)	128 (31.4)	119 (26.4)	105 (27.2)	
Smoking status, *n* (%)					0.034
Non smoker	298 (65.8)	289 (59.3)	316 (62.2)	292 (59.8)	
Ex-smoker	85 (18.8)	131 (26.9)	132 (26.0)	117 (24.0)	
Current smoker	70 (15.5)	67 (13.8)	60 (11.8)	79 (16.2)	
Physical activity, *n* (%)					0.585
Low	85 (21.5)	87 (19.1)	75 (16.4)	82 (19.5)	
Medium	190 (48.0)	218 (47.9)	234 (51.3)	214 (50.8)	
High	121 (30.6)	150 (33.0)	147 (32.2)	125 (29.7)	
Alcohol intake, *n* (%)					<0.001
No	110 (24.2)	108 (22.2)	97 (19.1)	60 (12.3)	
Moderate	324 (71.4)	319 (65.5)	290 (57.1)	273 (55.9)	
Regular	20 (4.4)	60 (12.3)	121 (23.8)	155 (31.8)	
Age (years), mean (SD)	47.4 (19.3)	48.7 (18.2)	48.0 (16.0)	49.5 (16.9)	0.296
Sodium (mg/day), mean (SD)	2669 (864.2)	2868 (1074.2)	2793.2 (1174.3)	3093.8 (1224.1)	<0.001
Potassium(mg/day), mean (SD)	2892 (848.8)	3471 (1027.1)	3892.1(1306.1)	4392.4 (1713.6)	<0.001
Magnesium (mg/day), mean (SD)	311.1 (87.9)	382.5 (109.5)	416.2 (128.6)	467.5 (172.7)	<0.001
Calcium (mg/day), mean (SD)	690.5 (258.3)	755.6 (264.7)	808.3 (331.0)	951.6 (400.5)	<0.001
High adherence to the Mediterranean diet (high)	40 (14.5)	102 (37.1)	82 (29.8)	51 (18.5)	<0.001

**Table 2:** Association between total, main classes, and individual phenolic acids and hypertension.

	Phenolic Acid Intake				
	Q1	Q2	Q3	Q4	*p* for Trend
Phenolic acids, mean (range), mg/day	112.12 (19.52, 156.19)	204.05 (156.28, 248.85)	311.00 (248.93, 385.09)	807.81 (386.07, 8361.62)	
No. of cases	224	278	257	217	
Model 1, OR (95% CI) ^a^	1	1.27 (0.95, 1.70)	0.97 (0.73, 1.29)	0.72 (0.52, 0.98)	
Model 2, OR (95% CI) ^b^	1	1.16 (0.80, 1.67)	0.86 (0.59, 1.24)	0.74 (0.48, 1.13)	
Model 3, OR (95% CI) ^c^	1	1.08 (0.73, 1.60)	0.74 (0.49, 1.10)	0.65 (0.43, 0.98)	0.056
Hydroxybenzoic acids, mean (range), mg/day	12.81 (0.00, 47.36)	64.18 (47.73, 81.36)	136.38 (81.50, 258.37)	617.46 (258.59, 8265.42)	
No. of cases	231	282	241	222	
Model 1, OR (95% CI) ^a^	1	1.49 (1.10, 2.00)	1.13 (0.84, 1.52)	0.85 (0.63, 1.15)	
Model 2, OR (95% CI) ^b^	1	1.25 (0.89, 1.76)	0.78 (0.53, 1.13)	0.93 (0.64, 1.35)	
Model 3, OR (95% CI) ^c^	1	1.21 (0.84, 1.75)	0.79 (0.52, 1.19)	0.93 (0.62, 1.40)	0.237
Hydroxycinammic acid, mean (range), mg/day	62.18 (14.72, 84.14)	106.72 (84.14, 128.85)	156.65 (128.87, 191.00)	271.05 (191.06, 836.62)	
No. of cases	223	261	267	225	
Model 1, OR (95% CI) ^a^	1	1.17 (0.87, 1.57)	1.01 (0.75, 1.37)	0.85 (0.62, 1.17)	
Model 2, OR (95% CI) ^b^	1	0.96 (0.66, 1.39)	0.74 (0.50, 1.09)	0.71 (0.46, 1.11)	
Model 3, OR (95% CI) ^c^	1	0.82 (0.55, 1.21)	0.61 (0.40, 0.94)	0.52 (0.32, 0.85)	<0.001
Hydroxyphenylacetic acid, mean (range), mg/day	0.03 (0.00, 0.09)	0.15 (0.09, 0.23)	0.36 (0.23, 0.48)	1.31 (0.48, 13.52)	
No. of cases	226	264	248	238	
Model 1, OR (95% CI) ^a^	1	1.34 (1.00, 1.81)	1.18 (0.88, 1.60)	1.00 (0.73, 1.36)	
Model 2, OR (95% CI) ^b^	1	1.15 (0.80, 1.65)	0.98 (0.68, 1.42)	0.70 (0.46, 1.08)	
Model 3, OR (95% CI) ^c^	1	0.95 (0.65, 1.39)	0.85 (0.57, 1.27)	0.61 (0.39, 0.97)	<0.001
Caffeic acid, mean (range), mg/day	0.41 (0.00, 0.60)	0.79 (0.60, 1.00)	1.43 (1.00, 2.10)	4.01 (2.11, 10.33)	
No. of cases	243	229	250	254	
Model 1, OR (95% CI) ^a^	1	0.71 (0.52, 0.95)	0.82 (0.61, 1.10)	0.88 (0.64, 1.22)	
Model 2, OR (95% CI) ^b^	1	0.86 (0.59, 1.26)	0.92 (0.62, 1.37)	0.70 (0.40, 1.22)	
Model 3, OR (95% CI) ^c^	1	0.99 (0.67, 1.50)	0.94 (0.61, 1.46)	0.83 (0.46, 1.50)	0.041
Cinnamic acid, mean (range), mg/day	0.02 (0.00, 0.08)	0.12 (0.08, 0.19)	0.28 (0.20, 0.43)	1.17 (0.43, 13.62)	
No. of cases	246	239	220	271	
Model 1, OR (95% CI) ^a^	1	0.83 (0.61, 1.12)	0.88 (0.65, 1.20)	0.90 (0.67, 1.22)	
Model 2, OR (95% CI) ^b^	1	0.70 (0.49, 0.99)	0.58 (0.40, 0.83)	0.81 (0.56, 1.16)	
Model 3, OR (95% CI) ^c^	1	0.68 (0.47, 0.98)	0.56 (0.38, 0.84)	0.74 (0.50, 1.10)	0.631
Vanillic acid, mean (range), mg/day	0.05 (0.00, 0.08)	0.14 (0.08, 0.22)	0.35 (0.22, 0.54)	0.99 (0.54, 5.02)	
No. of cases	239	263	238	236	
Model 1, OR (95% CI) ^a^	1	1.33 (0.98, 1.79)	0.95 (0.70, 1.28)	0.93 (0.68, 1.27)	
Model 2, OR (95% CI) ^b^	1	1.26 (0.87, 1.83)	0.88 (0.60, 1.30)	0.75 (0.47, 1.18)	
Model 3, OR (95% CI) ^c^	1	1.11 (0.75, 1.64)	0.91 (0.60, 1.39)	0.75 (0.46, 1.23)	0.427
Ferulic acid, mean (range), mg/day	0.55 (0.00, 0.93)	1.38 (0.93, 1.85)	2.69 (1.85, 4.04)	6.96 (4.05, 20.77)	
No. of cases	244	248	279	205	
Model 1, OR (95% CI) ^a^	1	0.93 (0.69, 1.25)	1.19 (0.88, 1.60)	0.69 (0.50, 0.95)	
Model 2, OR (95% CI) ^b^	1	1.14 (0.79, 1.64)	1.44 (1.01, 2.07)	0.78 (0.51, 1.18)	
Model 3, OR (95% CI) ^c^	1	1.25 (0.84, 1.86)	1.76 (1.18, 2.61)	0.82 (0.52, 1.30)	0.398

^a^ Model 1 adjusted for age (years, continuous), energy intake (kcal/day, continuous); ^b^ Model 2 = Model 1 + body mass index, smoking status (smokers, ex-smokers, non-smokers), alcohol consumption (0 g/day, <12 g/day, ≥12 g/day), physical activity level (low, medium, high), educational level (low, medium, high), occupational level (unemployed, low, medium, high), menopausal status (in women), fiber, sodium, potassium, magnesium, and calcium intake; ^c^ Model 3 = Model 2 + adherence to the Mediterranean diet. OR (odds ratio); CI (confidence interval).

**Table 3:** Association between major food sources of phenolic acids and hypertension.

	Food group intake, OR (95% CI) ^a^			
	Q1	Q2	Q3	Q4
Coffee ^b^	1	0.85 (0.51, 1.44)	1.03 (0.66, 1.62)	0.70 (0.45, 1.08)
Nuts ^c^	1	1.19 (0.90, 1.59)	1.07 (0.69, 1.67)	1.24 (0.73, 2.12)
Tea ^d^	1	1.12 (0.86, 1.47)	0.75 (0.48, 1.17)	0.45 (0.17, 1.14)
Olive oil ^e^	1	0.53 (0.22, 1.23)	0.50 (0.22, 1.10)	0.63 (0.29, 1.37)
Red wine ^f^	1	0.75 (0.53, 1.06)	1.13 (0.52, 2.45)	0.74 (0.24, 2.25)
White wine ^f^	1	0.72 (0.54, 0.95)	1.25 (0.38, 4.11)	-
Beer ^g^	1	0.83 (0.58, 1.20)	0.51 (0.32, 0.81)	0.32 (0.15, 0.68)

^a^ OR adjusted for sex, age (years, continuous), energy intake (kcal/day, continuous), smoking status (smokers, ex-smokers, non-smokers), alcohol consumption (0 g/day, <12 g/day, ≥12 g/day), physical activity level (low, medium, high), educational level (low, medium, high), occupational level (unemployed, low, medium, high), menopausal status (in women), fiber, sodium and potassium intake; ^b^ categories of coffee intake were as follow: Q1, none; Q2, <25 mL/day; Q3, 25–50 mL/day; Q4, >50 mL/day; ^c^ categories of nuts intake were as follow: Q1, none; Q2, <28 g/day; Q3, 28–56 g/day; Q4, >56 g/day; ^d^ categories of tea intake were as follow: Q1, none; Q2, <250 mL/day; Q3, 250–500 mL/day; Q4, >500 mL/day; ^e^ categories of olive oil intake were as follow: Q1, <2.5 mL/day; Q2, 2.5–4 mL/day; Q3, 4.5–9 mL/day; Q4, >9 mL/day; ^f^ categories of wine intake were as follow: Q1, none; Q2, <100 mL/day; Q3, 100–250 mL/day; Q4, >250 mL/day; ^g^ categories of beer intake were as follow: Q1, none; Q2, <100 mL/day; Q3, 100–300 mL/day; Q4, >300 mL/day.

## Supplementary Materials

The following are available online at www.mdpi.com/2072-6643/9/10/1069/s1, Supplementary material (Table S1. Dietary intake of major micro- and macro-nutrients by quartiles of dietary phenolic acids in the MEAL study sample (*n* = 1936); Table S2. Association between total, main classes, and individual phenolic acids and hypertension, separately for men and women).

## References

[1] Maghsoudi Z., Ghiasvand R., Salehi-Abargouei A. (2016). Empirically derived dietary patterns and incident type 2 diabetes mellitus: A systematic review and meta-analysis on prospective observational studies. Public Health Nutr..

[2] Rezagholizadeh F., Djafarian K., Khosravi S., Shab-Bidar S. (2017). A posteriori healthy dietary patterns may decrease the risk of central obesity: Findings from a systematic review and meta-analysis. Nutr. Res..

[3] Wang C.J., Shen Y.X., Liu Y. (2016). Empirically derived dietary patterns and hypertension likelihood: A meta-analysis. Kidney Blood Press Res..

[4] Beidokhti M.N., Jager A.K. (2017). Review of antidiabetic fruits, vegetables, beverages, oils and spices commonly consumed in the diet. J. Ethnopharmacol..

[5] Grosso G., Godos J., Galvano F., Giovannucci E.L. (2017). Coffee, caffeine, and health outcomes: An umbrella review. Annu. Rev. Nutr..

[6] Schwingshackl L., Hoffmann G., Missbach B., Stelmach-Mardas M., Boeing H. (2017). An umbrella review of nuts intake and risk of cardiovascualr disease. Curr. Pharm. Des..

[7] De Gaetano G., Costanzo S., Di Castelnuovo A., Badimon L., Bejko D., Alkerwi A., Chiva-Blanch G., Estruch R., La Vecchia C., Panico S. (2016). Effects of moderate beer consumption on health and disease: A consensus document. Nutr. Metab. Cardiovasc. Dis..

[8] Grosso G., Yang J., Marventano S., Micek A., Galvano F., Kales S.N. (2015). Nut consumption on all-cause, cardiovascular, and cancer mortality risk: A systematic review and meta-analysis of epidemiologic studies. Am. J. Clin. Nutr..

[9] Grosso G., Micek A., Godos J., Sciacca S., Pajak A., Martinez-Gonzalez M.A., Giovannucci E.L., Galvano F. (2016). Coffee consumption and risk of all-cause, cardiovascular, and cancer mortality in smokers and non-smokers: A dose-response meta-analysis. Eur. J. Epidemiol..

[10] Costanzo S., Di Castelnuovo A., Donati M.B., Iacoviello L., de Gaetano G. (2010). Alcohol consumption and mortality in patients with cardiovascular disease: A meta-analysis. J. Am. Coll. Cardiol..

[11] Rienks J., Barbaresko J., Nothlings U. (2017). Association of polyphenol biomarkers with cardiovascular disease and mortality risk: A systematic review and meta-analysis of observational studies. Nutrients.

[12] Grosso G., Micek A., Godos J., Pajak A., Sciacca S., Galvano F., Giovannucci E.L. (2017). Dietary flavonoid and lignan intake and mortality in prospective cohort studies: Systematic review and dose-response meta-analysis. Am. J. Epidemiol..

[13] Del Rio D., Costa L.G., Lean M.E., Crozier A. (2010). Polyphenols and health: What compounds are involved?. Nutr. Metab. Cardiovasc. Dis..

[14] Del Rio D., Rodriguez-Mateos A., Spencer J.P., Tognolini M., Borges G., Crozier A. (2013). Dietary (poly)phenolics in human health: Structures, bioavailability, and evidence of protective effects against chronic diseases. Antioxid. Redox Signal..

[15] Sohrab G., Hosseinpour-Niazi S., Hejazi J., Yuzbashian E., Mirmiran P., Azizi F. (2013). Dietary polyphenols and metabolic syndrome among Iranian adults. Int. J. Food Sci. Nutr..

[16] Miranda A.M., Steluti J., Fisberg R.M., Marchioni D.M. (2016). Association between polyphenol intake and hypertension in adults and older adults: A population-based study in Brazil. PLoS ONE.

[17] Grosso G., Stepaniak U., Micek A., Stefler D., Bobak M., Pajak A. (2017). Dietary polyphenols are inversely associated with metabolic syndrome in polish adults of the hapiee study. Eur. J. Nutr..

[18] Grosso G., Stepaniak U., Micek A., Kozela M., Stefler D., Bobak M., Pajak A. (2017). Dietary polyphenol intake and risk of hypertension in the polish arm of the hapiee study. Eur. J. Nutr..

[19] Grosso G., Marventano S., D’Urso M., Mistretta A., Galvano F. (2017). The Mediterranean healthy eating, ageing, and lifestyle (meal) study: Rationale and study design. Int. J. Food Sci. Nutr..

[20] Mistretta A., Marventano S., Platania A., Godos J., Galvano F., Grosso G. (2017). Metabolic profile of the Mediterranean healthy eating, lifestyle and aging (meal) study cohort. Mediterr. J. Nutr. Metab..

[21] Craig C.L., Marshall A.L., Sjostrom M., Bauman A.E., Booth M.L., Ainsworth B.E., Pratt M., Ekelund U., Yngve A., Sallis J.F. (2003). International physical activity questionnaire: 12-country reliability and validity. Med. Sci. Sports Exerc..

[22] Buscemi S., Rosafio G., Vasto S., Massenti F.M., Grosso G., Galvano F., Rini N., Barile A.M., Maniaci V., Cosentino L. (2015). Validation of a food frequency questionnaire for use in italian adults living in sicily. Int. J. Food Sci. Nutr..

[23] Marventano S., Mistretta A., Platania A., Galvano F., Grosso G. (2016). Reliability and relative validity of a food frequency questionnaire for italian adults living in sicily, southern Italy. Int. J. Food Sci. Nutr..

[24] Istituto Nazionale di Ricerca per gli Alimenti e la Nutrizione (2009). Tabelle di Composizione Degli Alimenti.

[25] Godos J., Marventano S., Mistretta A., Galvano F., Grosso G. (2017). Dietary sources of polyphenols in the Mediterranean healthy eating, aging and lifestyle (meal) study cohort. Int. J. Food Sci. Nutr..

[26] Neveu V., Perez-Jiménez J., Vos F., Crespy V., du Chaffaut L., Mennen L., Knox C., Eisner R., Cruz J., Wishart D. (2010). Phenol-explorer: An online comprehensive database on polyphenol contents in foods. Database.

[27] Rothwell J.A., Perez-Jimenez J., Neveu V., Medina-Remon A., M’Hiri N., Garcia-Lobato P., Manach C., Knox C., Eisner R., Wishart D.S. (2013). Phenol-explorer 3.0: A major update of the phenol-explorer database to incorporate data on the effects of food processing on polyphenol content. Database.

[28] Bognar A. (2002). Tables on Weight Yield of Food and Retention Factors of Food Constituents for the Calculation of Nutrient Composition of Cooked Foods (Dishes).

[29] Willett W. (1998). Reproducibility and validity of food frequency questionnaire. Nutritional Epidemiology.

[30] Buscemi S., Marventano S., Antoci M., Cagnetti A., Castorina G., Galvano F., Marranzano M., Mistretta A. (2016). Coffee and metabolic impairment: An updated review of epidemiological studies. NFS J..

[31] Bolling B.W., Chen C.Y., McKay D.L., Blumberg J.B. (2011). Tree nut phytochemicals: Composition, antioxidant capacity, bioactivity, impact factors. A systematic review of almonds, brazils, cashews, hazelnuts, macadamias, pecans, pine nuts, pistachios and walnuts. Nutr. Res. Rev..

[32] Barbour J.A., Howe P.R., Buckley J.D., Bryan J., Coates A.M. (2014). Nut consumption for vascular health and cognitive function. Nutr. Res. Rev..

[33] Zhao Y., Wang J., Ballevre O., Luo H., Zhang W. (2012). Antihypertensive effects and mechanisms of chlorogenic acids. Hypertens. Res..

[34] Ochiai R., Sugiura Y., Otsuka K., Katsuragi Y., Hashiguchi T. (2015). Coffee bean polyphenols ameliorate postprandial endothelial dysfunction in healthy male adults. Int. J. Food Sci. Nutr..

[35] Rahman I., Biswas S.K., Kirkham P.A. (2006). Regulation of inflammation and redox signaling by dietary polyphenols. Biochem. Pharmacol..

[36] Marventano S., Salomone F., Godos J., Pluchinotta F., Del Rio D., Mistretta A., Grosso G. (2016). Coffee and tea consumption in relation with non-alcoholic fatty liver and metabolic syndrome: A systematic review and meta-analysis of observational studies. Clin. Nutr..

[37] Grosso G., Micek A., Godos J., Pajak A., Sciacca S., Bes-Rastrollo M., Galvano F., Martinez-Gonzalez M.A. (2017). Long-term coffee consumption is associated with decreased incidence of new-onset hypertension: A dose-response meta-analysis. Nutrients.

[38] Briasoulis A., Agarwal V., Messerli F.H. (2012). Alcohol consumption and the risk of hypertension in men and women: A systematic review and meta-analysis. J. Clin. Hypertens..

[39] Poli A., Marangoni F., Avogaro A., Barba G., Bellentani S., Bucci M., Cambieri R., Catapano A.L., Costanzo S., Cricelli C. (2013). Moderate alcohol use and health: A consensus document. Nutr. Metab. Cardiovasc. Dis..

[40] Giacosa A., Adam-Blondon A.F., Baer-Sinnott S., Barale R., Bavaresco L., Di Gaspero G., Dugo L., Ellison R.C., Gerbi V., Gifford D. (2012). Alcohol and wine in relation to cancer and other diseases. Eur. J. Cancer Prev..

[41] Di Castelnuovo A., Costanzo S., Bagnardi V., Donati M.B., Iacoviello L., de Gaetano G. (2006). Alcohol dosing and total mortality in men and women: An updated meta-analysis of 34 prospective studies. Arch. Intern. Med..

[42] Arranz S., Chiva-Blanch G., Valderas-Martinez P., Medina-Remon A., Lamuela-Raventos R.M., Estruch R. (2012). Wine, beer, alcohol and polyphenols on cardiovascular disease and cancer. Nutrients.

[43] Adamsson V., Reumark A., Fredriksson I.B., Hammarstrom E., Vessby B., Johansson G., Riserus U. (2011). Effects of a healthy nordic diet on cardiovascular risk factors in hypercholesterolaemic subjects: A randomized controlled trial (nordiet). J. Intern. Med..

[44] Esposito K., Marfella R., Ciotola M., Di Palo C., Giugliano F., Giugliano G., D’Armiento M., D’Andrea F., Giugliano D. (2004). Effect of a Mediterranean-style diet on endothelial dysfunction and markers of vascular inflammation in the metabolic syndrome: A randomized trial. JAMA.

[45] Thomazella M.C., Goes M.F., Andrade C.R., Debbas V., Barbeiro D.F., Correia R.L., Marie S.K., Cardounel A.J., daLuz P.L., Laurindo F.R. (2011). Effects of high adherence to Mediterranean or low-fat diets in medicated secondary prevention patients. Am. J. Cardiol..

[46] Toledo E., Hu F.B., Estruch R., Buil-Cosiales P., Corella D., Salas-Salvado J., Covas M.I., Aros F., Gomez-Gracia E., Fiol M. (2013). Effect of the Mediterranean diet on blood pressure in the predimed trial: Results from a randomized controlled trial. BMC Med..

[47] Storniolo C.E., Casillas R., Bullo M., Castaner O., Ros E., Saez G.T., Toledo E., Estruch R., Ruiz-Gutierrez V., Fito M. (2017). A Mediterranean diet supplemented with extra virgin olive oil or nuts improves endothelial markers involved in blood pressure control in hypertensive women. Eur. J. Nutr..

[48] Grosso G., Estruch R. (2016). Nut consumption and age-related disease. Maturitas.

[49] Tresserra-Rimbau A., Medina-Remon A., Lamuela-Raventos R.M., Bullo M., Salas-Salvado J., Corella D., Fito M., Gea A., Gomez-Gracia E., Lapetra J. (2015). Moderate red wine consumption is associated with a lower prevalence of the metabolic syndrome in the predimed population. Br. J. Nutr..

[50] Giacosa A., Barale R., Bavaresco L., Faliva M.A., Gerbi V., La Vecchia C., Negri E., Opizzi A., Perna S., Pezzotti M. (2016). Mediterranean way of drinking and longevity. Crit. Rev. Food Sci. Nutr..

[51] Gepner Y., Henkin Y., Schwarzfuchs D., Golan R., Durst R., Shelef I., Harman-Boehm I., Spitzen S., Witkow S., Novack L. (2016). Differential effect of initiating moderate red wine consumption on 24-h blood pressure by alcohol dehydrogenase genotypes: Randomized trial in type 2 diabetes. Am. J. Hypertens..

[52] Bawaked R.A., Schroder H., Ribas-Barba L., Cardenas G., Pena-Quintana L., Perez-Rodrigo C., Fito M., Serra-Majem L. (2017). Dietary flavonoids of spanish youth: Intakes, sources, and association with the Mediterranean diet. PeerJ.

[53] Bonaccio M., Pounis G., Cerletti C., Donati M.B., Iacoviello L., de Gaetano G., Investigators M.-S.S. (2017). Mediterranean diet, dietary polyphenols and low grade inflammation: Results from the moli-sani study. Br. J. Clin. Pharmacol..

[54] Medina-Remon A., Casas R., Tressserra-Rimbau A., Ros E., Martinez-Gonzalez M.A., Fito M., Corella D., Salas-Salvado J., Lamuela-Raventos R.M., Estruch R. (2017). Polyphenol intake from a Mediterranean diet decreases inflammatory biomarkers related to atherosclerosis: A substudy of the predimed trial. Br. J. Clin. Pharmacol..

[55] Zamora-Ros R., Knaze V., Lujan-Barroso L., Romieu I., Scalbert A., Slimani N., Hjartaker A., Engeset D., Skeie G., Overvad K. (2013). Differences in dietary intakes, food sources and determinants of total flavonoids between Mediterranean and non-Mediterranean countries participating in the european prospective investigation into cancer and nutrition (epic) study. Br. J. Nutr..

[56] Godos J., Federico A., Dallio M., Scazzina F. (2016). Mediterranean diet and nonalcoholic fatty liver disease: Molecular mechanisms of protection. Int. J. Food Sci. Nutr..

[57] Godos J., Zappala G., Bernardini S., Giambini I., Bes-Rastrollo M., Martinez-Gonzalez M. (2017). Adherence to the Mediterranean diet is inversely associated with metabolic syndrome occurrence: A meta-analysis of observational studies. Int. J. Food Sci. Nutr..

[58] Grosso G., Mistretta A., Marventano S., Purrello A., Vitaglione P., Calabrese G., Drago F., Galvano F. (2014). Beneficial effects of the Mediterranean diet on metabolic syndrome. Curr. Pharm. Des..

[59] Grosso G., Marventano S., Yang J., Micek A., Pajak A., Scalfi L., Galvano F., Kales S.N. (2017). A comprehensive meta-analysis on evidence of Mediterranean diet and cardiovascular disease: Are individual components equal?. Crit. Rev. Food Sci. Nutr..

[60] Buscemi S., Marventano S., Castellano S., Nolfo F., Rametta S., Giorgianni G., Matalone M., Marranzano M., Mistretta A. (2016). Role of anthropometric factors, self-perception, and diet on weight misperception among young adolescents: A cross-sectional study. Eat. Weight Disord..

[61] Mistretta A., Marventano S., Antoci M., Cagnetti A., Giogianni G., Nolfo F., Rametta S., Pecora G., Marranzano M. (2017). Mediterranean diet adherence and body composition among southern Italian adolescents. Obes. Res. Clin. Pract..

[62] La Verde M., Mulè S., Zappalà G., Privitera G., Maugeri G., Pecora F., Marranzano M. (2017). Higher adherence to the Mediterranean diet is inversely associated with having hypertension: Is low salt intake a mediating factor?. Int. J. Food Sci. Nutr..

[63] Godos J., Rapisarda G., Marventano S., Galvano F., Mistretta A., Grosso G. (2017). Association between polyphenol intake and adherence to the Mediterranean diet in sicily, southern Italy. NFS J..

[64] Grosso G., Pajak A., Mistretta A., Marventano S., Raciti T., Buscemi S., Drago F., Scalfi L., Galvano F. (2014). Protective role of the Mediterranean diet on several cardiovascular risk factors: Evidence from sicily, southern Italy. Nutr. Metab. Cardiovasc. Dis..

[65] Grosso G., Marventano S., Giorgianni G., Raciti T., Galvano F., Mistretta A. (2014). Mediterranean diet adherence rates in sicily, southern Italy. Public Health Nutr..

[66] Grosso G., Marventano S., Galvano F., Pajak A., Mistretta A. (2014). Factors associated with metabolic syndrome in a Mediterranean population: Role of caffeinated beverages. J. Epidemiol..

[67] Giacosa A., Barale R., Bavaresco L., Gatenby P., Gerbi V., Janssens J., Johnston B., Kas K., La Vecchia C., Mainguet P. (2013). Cancer prevention in europe: The Mediterranean diet as a protective choice. Eur. J. Cancer Prev..

