# Supplementary material for: Association between Dietary Phenolic Acids and Hypertension in a Mediterranean Cohort
Source: Nutrients. 2017 Sep 27;9(10):1069. doi: 10.3390/nu9101069 (PMC5691686; doi:10.3390/nu9101069)
Supplement: Supplementary file 1 [file nutrients-09-01069-s001.zip › nutrients-229872-supplementary.pdf]

Supplementary Table S1. Dietary intake of major micro- and macro-nutrients by quartiles of dietary phenolic acids in the MEAL study sample (n = 1,936).

	Phenolic acid intake			
	Q1	Q2	Q3	Q4
Energy intake (kcal/d), median (range)	1664.50 (1001, 3888)	1935 (1031, 4766)	1980 (1010, 5064)	2356 (1028, 5877)
Macronutrients				
Carbohydrates (g/d), median (range)	238.17 (100.17, 673.96)	276.52 (119.10, 639.65)	278.70 (115.51, 649.26)	308.98 (120.49, 897.75)
Fiber (g/d), median (range)	22.73 (6.62, 65.51)	27.82 (2.81, 78.07)	31.99 (7.60, 85.11)	35.03 (11.06, 150.50)
Protein (g/d), median (range)	69.88 (32.65, 155.77)	80.08 (29.26, 242.17)	81.80 (38.32, 180.06)	88.84 (37.16, 332.65)
Fat (g/d), median (range)				
Cholesterol (mg/d), median (range)	160.20 (63.02, 468.55)	166.45 (56.92, 837.53)	164.81 (18.17, 517.35)	206.67 (17.28, 921.06)
SFA, median (range)	18.67 (8.80, 61.79)	21.19 (8.61, 80.44)	21.53 (6.48, 74.25)	27.17 (8.05, 76.40)
MUFA, median (range)	21.25 (9.19, 46.20)	22.84 (11.01, 60.82)	23.84 (7.28, 62.08)	29.24 (14.39, 93.84)
PUFA, median (range)				
Micronutrients				
Vitamin A (Retinol), median (range)	621.44 (170.62, 1710.76)	738.88 (177.45, 2148.54)	844.28 (152.20, 2886.15)	947.77 (260.90, 4949.53)
Vitamin C (mg/d), median (range)	102.11 (5.28, 334.45)	126.64 (6.03, 488.80)	149.15 (12.20, 700.31)	171.71 (34.49, 1097.79)
Vitamin E (mg/d), median (range)	6.84 (1.85, 15.77)	7.64 (1.47, 21.89)	8.50 (3.50, 22.08)	9.62 (4.36, 31.93)
Vitamin B12, median (range)	5.15 (1.50, 13.39)	5.09 (1.49, 45.10)	5.62 (0.57, 27.49)	6.23 (2.23, 120.52)
Vitamin D, median (range)	3.69 (0.64, 15.77)	3.55 (0.59, 51.00)	4.36 (0.20, 24.90)	4.56 (0.50, 56.41)
Sodium (mg/d), median (range)	2556.32 (876.09, 5858.59)	2652.79 (796.59, 8858.45)	2692.49 (737.51, 9469.50)	2941.89 (829.25, 8646.17)
Potassium (mg/d), median (range)	2765.40 (872.14, 6209.83)	3389.39 (812.56, 7419.57)	3741.26 (1617.07, 8842.85)	4073.51 (1576.99, 15693.66)
Magnesium, median (range)	300.75 (147.59, 687.34)	369.72 (109.84, 778.09)	401.61 (170.38, 937.40)	439.31 (149.57, 1665.14)

Calcium, median (range)	639.66 (226.15, 2015.74)	726.55 (169.71, 2163.39)	737.29 (270.47, 1836.24)	848.99 (224.52, 3109.93)

Supplementary Table S2. Association between total, main classes and individual phenolic acids and hypertension, separately for men and women.

	Phenolic acids quartiles, men				Phenolic acids quartiles, women			
	Q1	Q2	Q3	Q4	Q1	Q2	Q3	Q4
Phenolic acids								
No. of cases	101	136	118	107	123	142	139	110
OR (95% CI)[a]	1	1.73 (1.10, 2.73)	0.98 (0.64, 1.51)	0.88 (0.55, 1.42)	1	1.45 (0.94, 2.25)	1.07 (0.70, 1.64)	0.75 (0.47, 1.19)
OR (95% CI)[b]	1	1.38 (0.78, 2.44)	0.98 (0.55, 1.72)	0.83 (0.43, 1.57)	1	1.08 (0.64, 1.80)	0.70 (0.41, 1.18)	0.59 (0.32,1.07)
OR (95% CI)[c]	1	1.18 (0.62, 2.22)	0.79 (0.41, 1.50)	0.60 (0.29,1.23)	1	1.01 (0.59, 1.73)	0.54 (0.31, 0.93)	0.59 (0.31, 1.11)
Hydroxybenzoic acids								
No. of cases	105	140	106	111	126	142	135	111
OR (95% CI)[a]	1	1.44 (0.91, 2.26)	0.74 (0.48, 1.16)	0.67 (0.43, 1.05)	1	1.67 (1.10, 2.55)	1.62 (1.06, 2.49)	0.96 (0.63, 1.47)
OR (95% CI)[b]	1	1.26 (0.74, 2.16)	0.64 (0.35, 1.17)	0.82 (0.46, 1.47)	1	1.48 (0.91, 2.42)	1.05 (0.61, 1.79)	1.04 (0.62, 1.77)
OR (95% CI)[c]	1	0.85 (0.48, 1.53)	0.37 (0.19, 0.73)	0.61 (0.32, 1.15)	1	1.42 (0.87, 2.33)	1.07 (0.61, 1.87)	1.11 (0.64, 1.90)
Hydroxycinammic acid								
No. of cases	106	118	132	106	117	143	135	119
OR (95% CI)[a]	1	1.22 (0.80, 1.86)	1.66 (1.06, 2.57)	1.23 (0.78, 1.94)	1	0.69 (0.40, 1.18)	0.42 (0.24, 0.72)	0.40 (0.21, 0.74)
OR (95% CI)[b]	1	1.12 (0.65, 1.91)	1.27 (0.71, 2.27)	1.22 (0.64, 2.33)	1	0.75 (0.44, 1.28)	0.45 (0.26, 0.78)	0.44 (0.24, 0.80)
OR (95% CI)[c]	1	0.75 (0.41, 1.38)	0.97 (0.52, 1.82)	0.67 (0.32, 1.42)	1	0.65 (0.37,1.13)	0.41 (0.23, 0.73)	0.35 (0.19, 0.66)

Hydroxyphenylacetic acid								
No. of cases	78	141	110	133	148	123	138	105
OR (95% CI)[a]	1	1.65 (1.04, 2.62)	0.83 (0.52, 1.31)	1.20 (0.74, 1.95)	1	0.97 (0.64, 1.47)	1.56 (1.03, 2.37)	0.84 (0.55, 1.30)
OR (95% CI)[b]	1	1.22 (0.67, 2.20)	0.71 (0.39, 1.28)	1.09 (0.55, 2.16)	1	0.87 (0.53, 1.42)	1.29 (0.76, 2.18)	0.46 (0.24, 0.87)
OR (95% CI)[c]	1	0.95 (0.49, 1.82)	0.52 (0.27, 1.00)	0.71 (0.33, 1.49)	1	0.77 (0.47, 1.28)	1.17 (0.67, 2.04)	0.44 (0.23, 0.84)
Caffeic acid								
No. of cases	110	112	123	117	133	117	127	137
OR (95% CI)[a]	1	0.63 (0.40, 0.98)	0.85 (0.54, 1.34)	0.70 (0.43, 1.15)	1	0.60 (0.39, 0.92)	0.67 (0.44, 1.03)	1.06 (0.67, 1.65)
OR (95% CI)[b]	1	0.87 (0.48, 1.60)	0.97 (0.51, 1.84)	0.46 (0.19, 1.06)	1	0.89 (0.52, 1.53)	0.89 (0.50, 1.55)	1.19 (0.58, 2.43)
OR (95% CI)[c]	1	0.77 (0.40, 1.49)	0.86 (0.44, 1.69)	0.20 (0.07, 0.52)	1	0.84 (0.49, 1.46)	0.77 (0.44, 1.34)	1.39 (0.65, 2.97)
Cinnamic acid								
No. of cases	98	99	118	147	148	140	102	124
OR (95% CI)[a]	1	0.60 (0.38, 0.95)	0.95 (0.59, 1.53)	0.95 (0.60, 1.51)	1	1.00 (0.66, 1.51)	0.78 (0.51, 1.21)	0.86 (0.56, 1.31)
OR (95% CI)[b]	1	0.39 (0.22, 0.69)	0.52 (0.28, 0.95)	0.82 (0.45, 1.48)	1	0.85 (0.53, 1.37)	0.50 (0.29, 0.84)	0.77 (0.46, 1.28)
OR (95% CI)[c]	1	0.35 (0.19, 0.65)	0.51 (0.26, 1.02)	0.73 (0.38, 1.41)	1	0.75 (0.46, 1.23)	0.45 (0.25, 0.78)	0.74 (0.43, 1.26)
Vanillic acid								
No. of cases	98	141	126	97	141	122	112	139

OR (95% CI)[a]	1	1.33 (0.85, 2.08)	0.74 (0.48, 1.16)	0.87 (0.53, 1.43)	1	1.14 (0.74, 1.74)	1.02 (0.67, 1.57)	0.99 (0.65, 1.50)
OR (95% CI)[b]	1	1.40 (0.78, 2.50)	0.69 (0.38, 1.26)	0.78 (0.38, 1.58)	1	0.95 (0.56, 1.60)	1.11 (0.65, 1.89)	0.81 (0.46, 1.42)
OR (95% CI)[c]	1	1.39 (0.73, 2.65)	0.61 (0.31, 1.17)	0.52 (0.24, 1.15)	1	0.92 (0.54, 1.59)	1.17 (0.68, 2.03)	0.84 (0.47, 1.49)
Ferulic acid								
No. of cases	1	126	148	77	133	122	131	128
OR (95% CI)[a]	1	0.89 (0.58, 1.38)	1.25 (0.81, 1.94)	0.75 (0.45, 1.25)	1	0.82 (0.54, 1.27)	0.91 (0.60, 1.40)	0.66 (0.43, 1.03)
OR (95% CI)[b]	1	1.38 (0.79, 2.39)	1.23 (0.71, 2.13)	0.89 (0.46, 1.73)	1	0.93 (0.53, 1.61)	1.41 (0.83, 2.39)	0.77 (0.43, 1.35)
OR (95% CI)[c]	1	1.39 (0.77, 2.49)	1.64 (0.89, 3.00)	0.96 (0.47, 1.98)	1	0.86 (0.49, 1.51)	1.64 (0.96, 2.81)	0.76 (0.43, 1.36)

[a] Model 1 adjusted for age (years, continuous), energy intake (kcal/d, continuous).

[b] Model 2 = Model 1 + body mass index, smoking status (smokers, ex-smokers, non-smokers), alcohol consumption (0 g/d,<12 g/d, ≥12 g/d), physical activity level (low, medium, high), educational level (low, medium, high), occupational level (unemployed, low, medium, high), menopausal status (in women), fiber, sodium, potassium, magnesium, and calcium intake.

[c] Model 3 = Model 2 + adherence to the Mediterranean diet.
